# TMEM88 Modulates Lipid Synthesis and Metabolism Cytokine by Regulating Wnt/β-Catenin Signaling Pathway in Non-Alcoholic Fatty Liver Disease

**DOI:** 10.3389/fphar.2021.798735

**Published:** 2022-01-04

**Authors:** Huan Zhou, Xingyu Zhu, Yan Yao, Yue Su, Jing Xie, Minhui Zhu, Cuixia He, Jiaxiang Ding, Yuanyuan Xu, Rongfang Shan, Ying Wang, Xiangdi Zhao, Yuzhou Ding, Bingyan Liu, Zhonghuan Shao, Yuanyuan Liu, Tao Xu, Yunqiu Xie

**Affiliations:** ^1^ National Drug Clinical Trial Center, The First Affiliated Hospital of Bengbu Medical College, Bengbu, China; ^2^ School of Pharmacy, Bengbu Medical College, Bengbu, China; ^3^ School of Public Foundation, Bengbu Medical University, Bengbu, China; ^4^ Inflammation and Immune Mediated Diseases Laboratory of Anhui Province, Anhui Institute of Innovative Drugs, School of Pharmacy, Anhui Medical University, Hefei, China

**Keywords:** lipid metabolism, AML-12 cells, Wnt/β-catenin, FFA, TMEM88

## Abstract

**Objective:** To clarify the molecular mechanism of TMEM88 regulating lipid synthesis and metabolism cytokine in NAFLD.

**Methods:** *In vivo*, NAFLD model mice were fed by a Methionine and Choline-Deficient (MCD) diet. H&E staining and immunohistochemistry experiments were used to analyze the mice liver tissue. RT-qPCR and Western blotting were used to detect the lipid synthesis and metabolism cytokine. *In vitro*, pEGFP-C1-TMEM88 and TMEM88 siRNA were transfected respectively in free fat acid (FFA) induced AML-12 cells, and the expression level of SREBP-1c, PPAR-α, FASN, and ACOX-1 were evaluated by RT-qPCR and Western blotting.

**Results:** The study found that the secretion of PPAR-α and its downstream target ACOX-1 were upregulated, and the secretion of SREBP-1c and its downstream target FASN were downregulated after transfecting with pEGFP-C1-TMEM88. But when TMEM88 was inhibited, the experimental results were opposite to the aforementioned conclusions. The data suggested that it may be related to the occurrence, development, and end of NAFLD. Additionally, the study proved that TMEM88 can inhibit Wnt/β-catenin signaling pathway. Meanwhile, TMEM88 can accelerate the apoptotic rate of FFA-induced AML-12 cells.

**Conclusion:** Overall, the study proved that TMEM88 takes part in regulating the secretion of lipid synthesis and metabolism cytokine through the Wnt/β-catenin signaling pathway in AML-12 cells. Therefore, TMEM88 may be involved in the progress of NAFLD. Further research will bring new ideas for the study of NAFLD.

## Introduction

Non-alcoholic fatty liver disease (NAFLD) is a disorder of liver lipid metabolism under non-alcoholic stimulation and excessive precipitation of liver cell fat, leading to fatty liver, non-alcoholic steatohepatitis (NASH), and other related diseases (Maurice and Manousou 2018; Geh et al., 2021). With the global obesity epidemic, the incidence of NAFLD continues to increase, and it has become the most common chronic liver disease on a global scale (Younossi 2019). The incidence of NAFLD in adults is about 17–33%, and the incidence in obese people is as high as 75%. Among them, the incidence of cirrhosis in NAFLD for 10 years is 25% (Borrelli et al., 2018; Maurice and Manousou 2018). Although there are many theories and hypotheses about the pathogenesis of NAFLD, they fail to cover the whole process of occurrence and development. A lot of evidence showed that it was related to the imbalance of lipid homeostasis caused by abnormal regulation of lipid metabolism (Buzzetti et al., 2016). The accumulation of triglycerides in liver parenchymal cells is the main feature of NAFLD. The main mechanism of hepatocyte fat deposition is the imbalance of triglyceride synthesis and output in the liver (Mashek 2021). Most of the sterol regulatory elements of transcription cytokine are also downstream target genes of the binding protein such as fatty acid synthase (FAS). They have also been involved in the synthesis of carbohydrate reactive element–binding protein (ChREBP) in the liver (Barbaro et al.). According to relevant experimental reports, the Wnt/β-catenin signaling pathway can regulate liver lipid anabolism by regulating other regulatory cytokines, such as SREBP-1c, FAS, and PPAR family (Xu et al., 2014; Seo et al., 2016).

Additionally, TMEM88 is a potential type 2 transmembrane protein generated by transcription and translation on chromosome 17p13.1. However, as far as the current research was concerned, the research on the related expression mechanism of this protein was still limited, and its potential application value is gradually receiving attention. Recent studies showed that β-catenin located in the cytoplasm was recognized by the phosphorylation site of GSK3β and phosphorylated and degraded, thereby inhibiting the classic Wnt/β-catenin signaling pathway. Research on TMEM88 indicated that TMEM88 had an inhibitory effect on the Wnt/β-catenin signaling pathway in HEK293 cells, and this impact can also mediate the development of human embryonic stem cells to cardiomyocytes. In addition, an experiment found that TMEM88 has a protective function on alcoholic liver fibrosis (Li et al., 2020). It proved that TMEM88 can play a certain regulatory role in the happening and progress of liver diseases. Therefore, this experiment further speculated that TMEM88 may mediate the Wnt/β-catenin signaling pathway to maintain lipid homeostasis and protect liver cells.

This research was devised to probe the metabolic function and molecular mechanisms of TMEM88 in NAFLD. *In vivo* study, the MCD-fed mice were used to study the mechanism of TMEM88 in NAFLD. FFA-induced AML-12 cells were used to observe the regulatory mechanism of TMEM88 *in vitro*. The results showed that TMEM88 can regulate lipid metabolism cytokine which provided a strategy for the diagnosis and treatment for NAFLD-related diseases.

## Materials and Methods

### Materials

Dulbecco’s modified Eagle medium was purchased from HyClone (Beijing, China). Lipofectamine™ 2000 and TRIzol were purchased from Invitrogen (Carlsbad, CA, United States). An ECL-Chemiluminescence kit was purchased from ThermoFisher Scientific (NYC, United States). An FITC Annexin V Apoptosis Detection Kit I was purchased from BestBio (Shanghai, China). Fetal bovine serum (FBS) and Opti-MEM were purchased from Gibco (Grand Island, NY, United States). The primers of TMEM88, SREBP-1c, PPAR-α, FASN, and ACOX-1 were produced by Sheng gong Biotechnology Company (Shanghai, China). TBST was purchased from Boster (Boster, China). Extraction buffer, PMSF, and RIPA lysis buffer were purchased from Beyotime (Hangzhou, China). β-catenin inhibitor FH535 (HY-15721) was purchased from MCE (Shanghai, China). Methionine- and choline-deficient diet (MCD) (TP 3006) and methionine- and choline-supplement diet (MCS) (TP 3006S) were purchased from Trophic (Nantong, China). Palmitoleic acid (P860713) and oleic acid (O815202) were purchased from Macklin (Shanghai, China). TMEM88 (sc135525) monoclonal antibody was purchased from Santa Cruz Biotechnology (CA, United States). Anti-β-actin (Cat: TA-09) monoclonal antibody was purchased from Santa Cruz Biotechnology (CA, United States). c-myc (2278T), cyclin D1 (2978T) monoclonal antibodies were purchased from Cell Signaling Technology (MA, United States). PPAR-α(DF6073) and SREBP-1c (AF6283) monoclonal antibodies were purchased from Affinity (Cincinnati, United States). FASN (ab128870) and ACOX-1 (ab32072) were purchased from Abcam (Cambridge, United Kingdom). Goat anti-mouse immunoglobulin (IgG) (ZB-2305) and goat anti-rabbit immunoglobulin (IgG) (ZB-2301) secondary antibodies were purchased from ZSGBBIO (Beijing, China).

### Construction of Non-Alcoholic Fatty Liver Disease Model

The mice used to construct the NAFLD model were 8-weeks-old male C57BL/6J mice. The animal trial procedure was approved by the Ethics Committee of Bengbu Medical College. All male mice were randomly divided into two groups (*n* = 10). Normal group mice were fed with MCS feed, experimental group mice were fed with MCD. After 8 weeks of continuous feeding, the mice were anesthetized and killed in the early morning of the last day. Blood and liver tissue were collected for further analysis.

### Cell Culture

AML-12 cells were nourished in DME/F12 medium containing 10% FBS and nurtured in a 37°C moist incubator and 5% CO_2_. Different times and concentrations of FFA were used to stimulate AML-12 cells.

### Oil Red

The AML-12 cells were divided into two groups. The experimental group was induced by FFA for 24 h, then ORO Fixative was used to fix AML-12 cells for 15–25 min, 60% isopropyl alcohol was used to wash the cells for 5 min, and the ORO Stain was used to stain the AML-12 cells, then stained for 10–15 min by airtight, and water was used to remove the staining solution. Then Mayer hematoxylin staining solution was used to counter-stain the cell nucleus for 1∼2 min, then discard the staining solution and wash with water 3 times, then microscope was used to observe the stain.

### Edu Staining

4% fixative was used to fixate the fresh liver tissues for 24 h, then the tissues were dehydrated with ethanol from high concentration to low concentration, and put in xylene. The treated tissues were embedded in paraffin and sectioned, and then xylene and gradient alcohol were used to dewax and dehydrate. Then HE was used to dye, gradient alcohol was used to dehydrate. After permeation with xylene for 5 min, the tissues were observed by microscope.

### siRNA and Plasmid Transfection

The AML-12 cells were seeded into 6-well plates and transfected according to previous research methods (Barbaro, Romito, and Alisi). After 6 h of transfection, the cells were cultured in DMF/F12 medium for 24 h, and the siRNA sequence used for transfection is as follows: F:5′-UCAUGUUAGGCUUCGGCUUTT-3'; R:5′-AAGCCGAAGCCUAACAUGATT -3'; negative control: F: 5′-UUC​UCC​GAA​CGU​GUC​ACG​UTT-3′, R:5′-ACGUG ACA​CUG​GCG​GAG​AAT​T-3'.

### Cell Viability Assays

Cell proliferation was analyzed by using CCK-8 assay. Cells were transferred into 96-well plates with a volume of 100 ul per well, with a cell density of 1 × 10^4^ cells, and placed in the incubator until they adhere to the wall. Different concentrations of FFA were used to induce cells for 24 h. Then 10 ul of CCK8 solution was added to each well and incubated for 3 h in the incubator. Then a microplate reader was used to measure the optical density (OD) at a wavelength of 490 nm, and the percent viability was calculated. The experimental data were repeated three times.

### Flow Cytometry

After transfection and FFA stimulation, AML-12 cells were lysed with trypsin for 2 min and then gently pipetted into a 1.5-ml EP tube for centrifugation. The supernatant was discarded, and cold PBS was added for washing and centrifugation, and the previous operation was repeated three times. Annexin V working solution was added to resuspend the cells and then PI staining was performed, and the solution was incubated for 10 min in the dark.

### RNA Isolation and RT-qPCR

TRIzol reagent and chloroform were used to extract RNA from AML-12 cells, then RNA was transferred in the water phase to another RNase-free centrifuge tube and isopropanol was added to precipitate, and the resulting RNA was dissolved in enzyme-free water. The first-strand cDNA synthesis kit was used for RNA reversing. GAPDH was used as an internal reference for detection in an RT-qPCR instrument. The primers used for PCR are shown in Table 1.

**TABLE 1:** Primer sequences for quantitative real-time reverse transcription polymerase chain reaction.

Gene	Primer pair	
TMEM88	F:5′-GCGCTAGCATGGCGGATGGCCCCAGG -3′	R:5′-GCGCGGCCGCTTAGGCTTCATTTGTCTTTTCTTCAG-3′
SREBP-1c	F:5′-GCAGCAACGGGACCATTCT -3′	R:5′- CCC​CAT​GAC​TAA​GTC​CTT​CAA​CT -3′
PPAR-α	F:5′- AAC​ATC​GAG​TGT​CGA​ATA​TGT​GG -3′	R:5′- CCG​AAT​AGT​TCG​CCG​AAA​GAA -3′
FASN	F:5′- GGA​GGT​GGT​GAT​AGC​CGG​TAT -3′	R:5′- TGG​GTA​ATC​CAT​AGA​GCC​CAG -3′
ACOX-1	F:5′- TAA​CTT​CCT​CAC​TCG​AAG​CCA -3′	F:5′- AGT​TCC​ATG​ACC​CAT​CTC​TGT​C -3′
β-actin	F:5′-GCCAACACAGTGCTGTCTGG-3′	R:5′-CTCAGGAGGAGCAATGATCTTG-3′

### Western Blotting

A new RIPA lysis buffer containing 1% PMSF was used to extract proteins from liver tissue and AML-12 cells. The BCA protein detection kit measured the protein concentration. The same amount of protein was resolved by SDS-PAGE then the PVDF membrane was used to transfer. After blocking in blocking buffer, the membrane was washed three times in TBS-Tween buffer. The membrane with the specific primary antibody was incubated in the primary antibody dilution buffer in a 4°C refrigerator for 12 h. Meanwhile, β-actin was used by mouse secondary antibody. TMEM88, SREBP-1c, PPAR-α, FASN, ACOX-1, and β-catenin were used by rabbit secondary antibody. After washing three times with TBST, the ECL chemiluminescence kit was used to detect the protein.

### Immunohistochemistry

Before dewaxing, the liver slices were placed in a constant temperature oven at 60°C for 1 h. The tissue slices were soaked in xylene and ethanol of different concentrations for hydration, and then the slices were immersed in a sodium citrate buffer. Then the antigen was repaired by high pressure. After cooling to room temperature, 3% H_2_O_2_ was added and allowed to stand for 15 min for inhibiting endogenous peroxidase activity and then 2% bovine serum albumin (BSA), TMEM88 (1:100) was added. And α-SMA (1: 100), the primary antibody was incubated at 4°C for 16 h. The expression was identified by using DAB color-developing solution. The sections were subsequently counterstained with hematoxylin for 5 min and then dehydrated in reduced concentration ethanol, dried, photographed, mounted, and stored.

### Statistical Analysis

All experimental data were repeated in triplicate. The difference between groups was analyzed by t-tests or one-way ANOVA and processed by SPSS 18.0 software. The data were presented as mean ± standard deviation (SD). If a *p*-value was less than 0.05, the data were considered statistically significant. If a *p*-value was less than 0.01, the data were considered statistically significant.

## Results

### TMEM88 was Downregulated in Liver Tissue of Non-Alcoholic Fatty Liver Disease Mice

**FIGURE 1:**
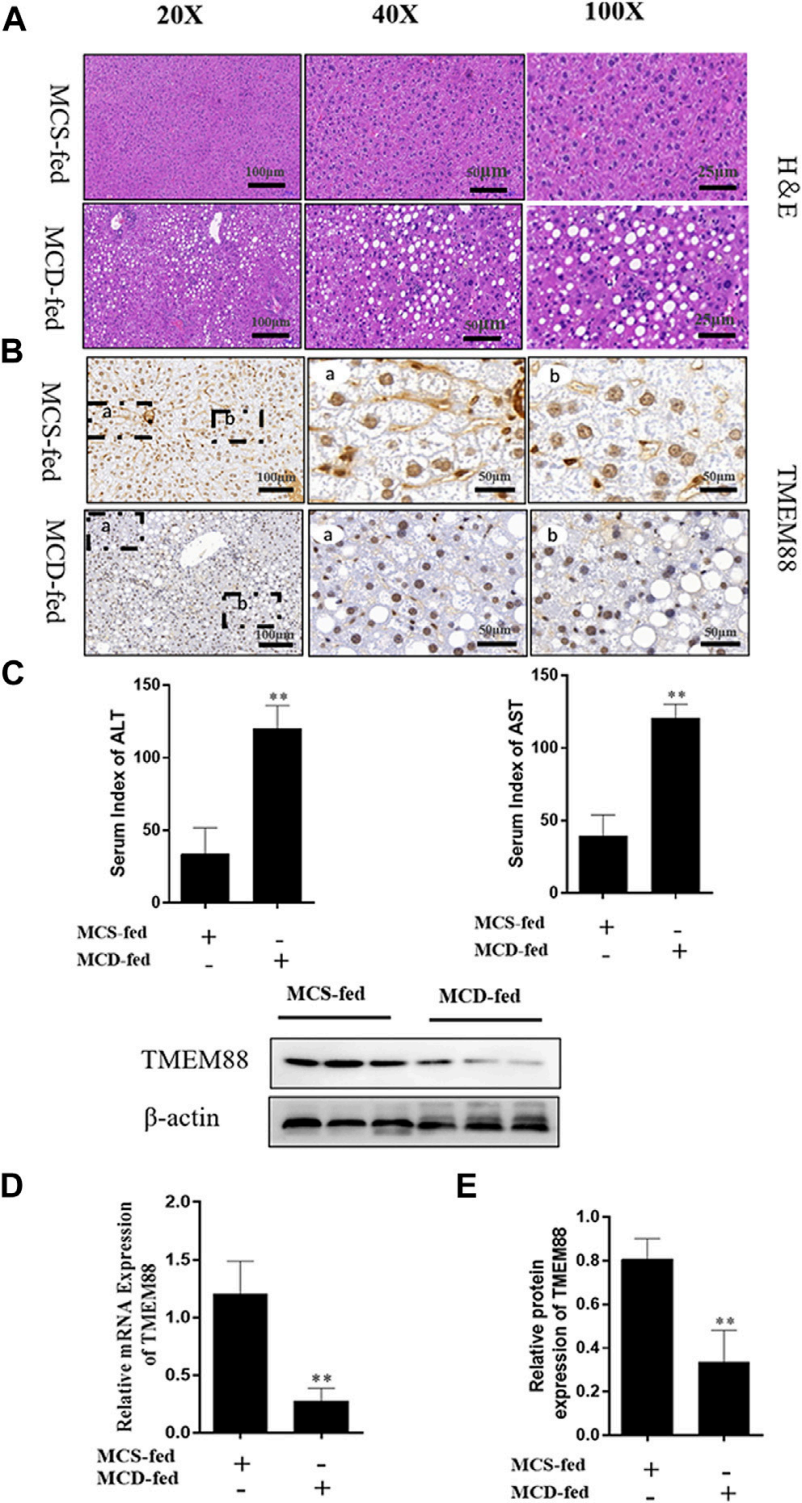
TMEM88 expression was suppressed in NAFLD model. **(A)** The H&E stain in liver tissues. Fat vacuoles and intercellular spaces dilatation were appeared in NAFLD liver tissues, while the normal liver tissues indicated that normal lobular architecture. **(B)** Immunohistochemistry indicated that the expression of TMEM88 was reduced in MCD-fed mice liver tissues of **(C)**. The serum levels of ALT and AST in the MCD-fed group were increased. **(D,E)** RNA and protein results showed that compared with the normal group, TMEM88 was inhibited in MCD-fed mice. (Data are represented by at least three independent mean ± SD, *p < 0.05, **p < 0.01 vs normal group).

To observe the pathological changes of NAFLD *in vivo*. In this experiment, all MCD-fed mice which developed fatty liver and injury caused by the imbalance of lipid metabolism. The H&E staining results proved that the the NAFLD liver of the MCD-fed group exhibited fatty vacuoles and inflammatory infiltration, the gap between cells was enlarged, and the liver cord was disordered (Figure 1A). Immunohistochemistry results indicated that the expression level of TMEM88 was reduced in the MCD-fed group (Figure 1B). In addition, the blood collected from the corner of the eye was used to test the serum. The result indicated that the expression level of ALT and AST was upregulated in the MCD-fed group (Figure 1C). The expression level of TMEM88 in NAFLD mice liver was tested to make sure that whether TMEM88 was involved in NAFLD. RT-qPCR and Western blotting results indicated that the expression level of TMEM88 was reduced in the MCD-fed group (Figures 1D,E).

**FIGURE 2:**
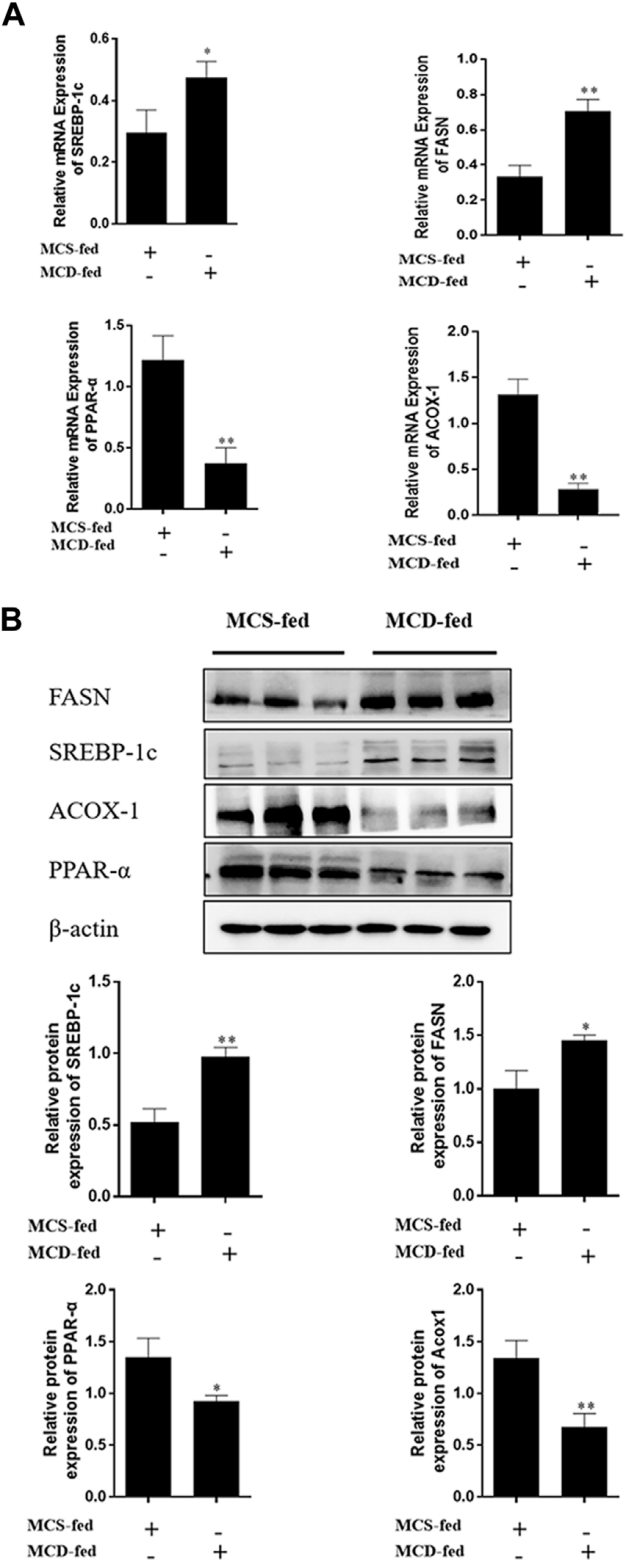
Expression level of lipid metabolism cytokine in MCD-fed mice. **(A,B)** The mRNA and protein expression levels of FASN and SERBP-1C were increased. PPAR-α and ACOX-1 expression were decreased in NAFLD liver tissues. (Data are represented by at least three independent mean ± SD, *p < 0.05, **p < 0.01 vs normal group).

SREBP-1c, PPAR-α, FASN, and ACOX-1 are representative lipid metabolism cytokines. The results suggested that expression of levels of PPAR-α and ACOX-1 were reduced, while SREBP-1c and FASN were upregulated in liver samples (Figures 2A,B).

### TMEM88 was Reduced in Free Fat Acid–Induced AML-12 Cells

**FIGURE 3:**
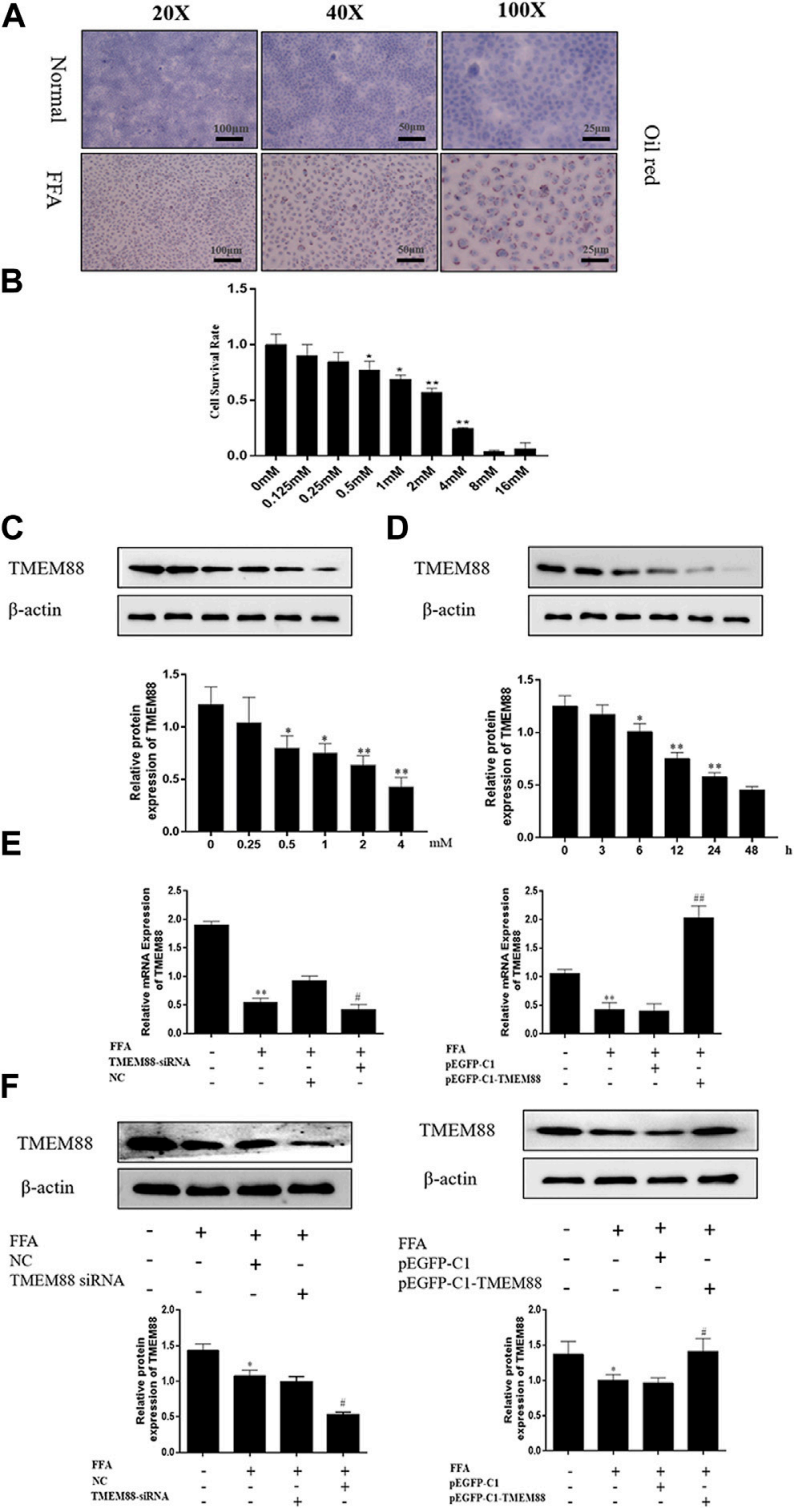
FFA inhibited TMEM88 expression in AML-12 cells. **(A)** The oil red stains in AML-12 cells and FFA-induced AML-12 cells. The results showed that fat granules appear in the FFA-induced cell model. **(B)** Viability of AML-12 cells at different FFA concentrations. **(C)** TMEM88 protein expression level was significantly reduced, after being induced with 2 mM FFA. **(D)** A significant reduction of the expression level of TMEM88 protein was significantly reduced after FFA was stimulated with 24 h. **(E)** TMEM88 mRNA expression levels in FFA-induced AML-12 cells after pEGFP-C1-TMEM88 and TMEM88 siRNA transfection. **(F)** TMEM88 protein expression levels in FFA-induced AML-12 cells after transfected with pEGFP-C1-TMEM88 and TMEM88 siRNA. (The data are represented by at least three independent mean ± SD, *p < 0.05, **p < 0.01 vs normal group. ^#^ *p* < 0.05, ^##^ *p* < 0.01 vs control group).

To observe the pathological changes of NAFLD *in vitro* and the expression level of TMEM88 in FFA-induced AML-12 cells. AML-12 cells were stimulated by different concentrations of FFA and at different times. First, the oil red staining results demonstrated that compared with the normal group, it can be seen that the FFA-induced group was successfully constructed (Figure 3A). Second, different concentrations of FFA were used to induce AML-12 cells for 24 h. It indicated a dose-dependent decrease in cell viability (Figure 3B). Third, the experimental results indicated that the expression level of TMEM88 continued to decrease with the increasing concentration of FFA stimulation in the experiment and reached a bottom protein level after 24 h of inducing by 2 mM FFA (Figure 3C). Fourth, the AML-12 cells were induced by 2 mM FFA in different hours to observe the expression level of TMEM88. Western blotting results proved that the TMEM88 protein level reached a valley when AML-12 cells were stimulated by FFA for 24 h (Figure 3D). Last, AML-12 cells were transfected with TMEM88siRNA and pEGFP-C1-TMEM88, respectively. The results showed that the TMEM88siRNA or pEGFP-C1-TMEM88, has been successfully transfected in AML-12 cells (Figures 3E,F).

### TMEM88 Promoted Lipid Metabolism in Free Fat Acid–Induced AML-12 Cells

**FIGURE 4:**
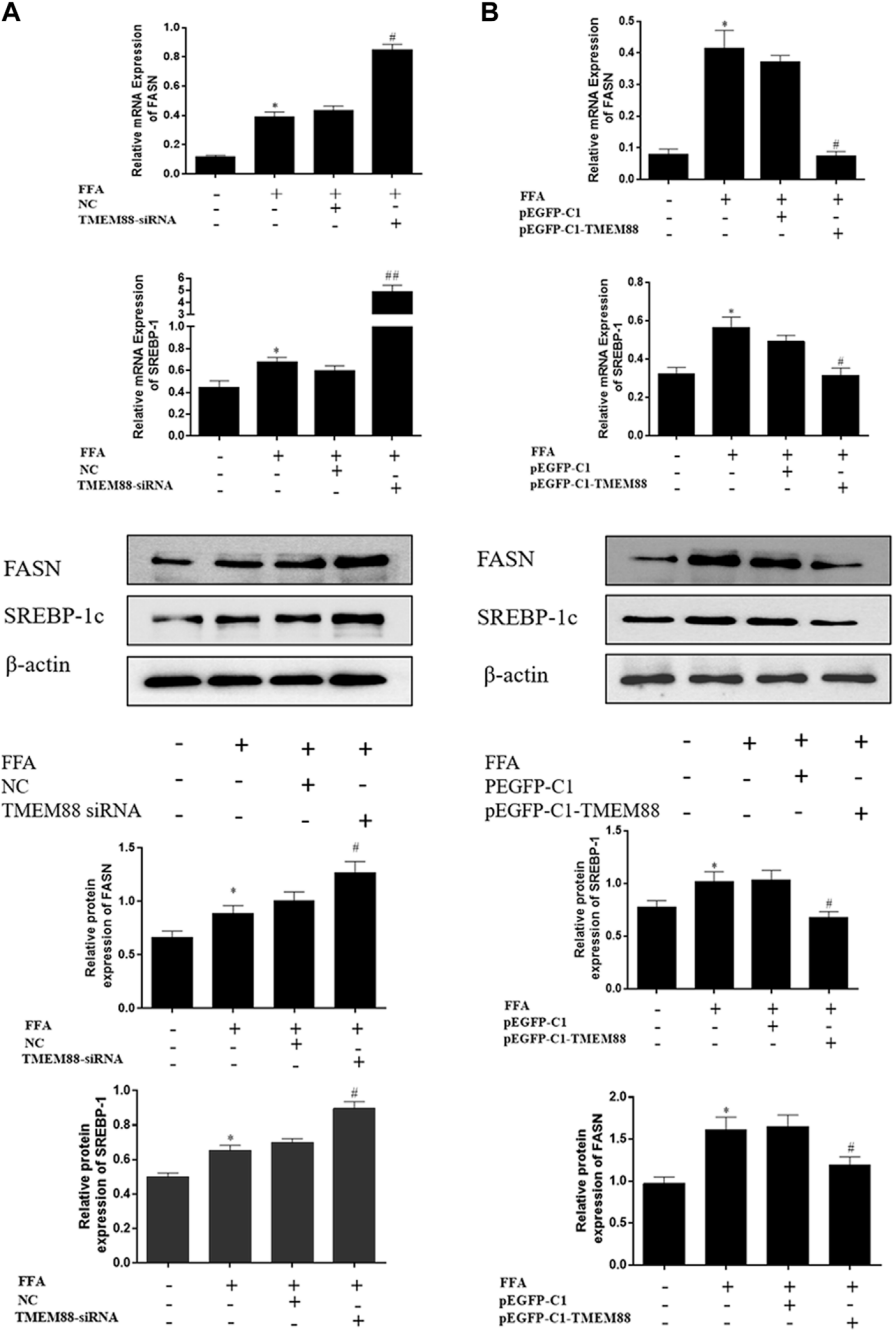
Silencing or overexpression of TMEM88 regulated the lipid metabolism in FFA-induced AML-12 cells **(A)** When TMEM88 was overexpressed, the expression levels of FASN and SREBP-1c decreased. In contrast, **(B)** When TMEM88 was silenced, the expression levels of FASN and SREBP-1c were upregulated. (Data are represented by at least three independent mean ± SD, *p < 0.05 vs normal group, ^#^ *p* < 0.05, ^##^ *p* < 0.01 vs control group).

**FIGURE 5:**
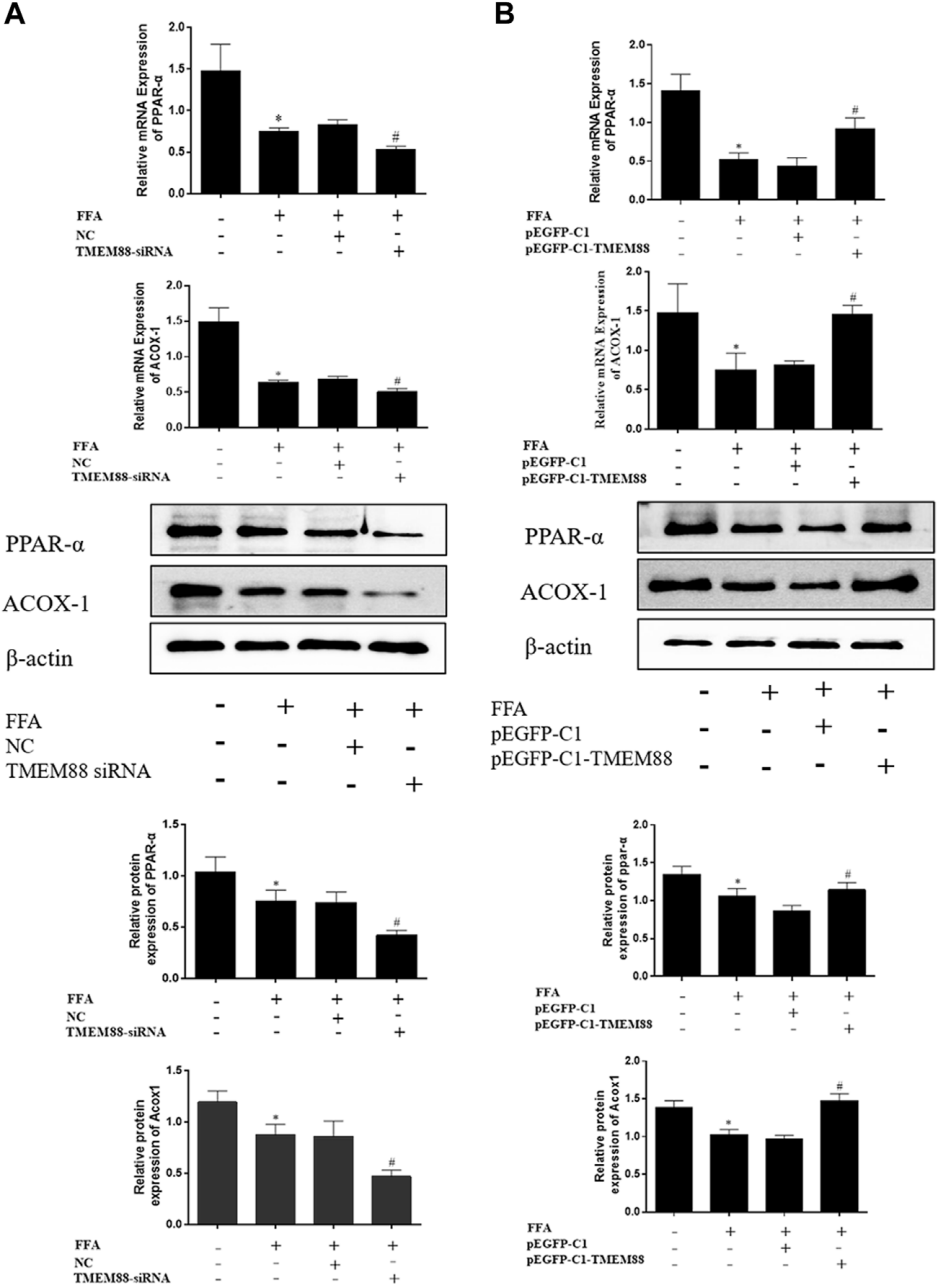
Silencing or overexpression of TMEM88 regulated the lipid metabolism in FFA-induced AML-12 cells. **(A)** The results of RT-qPCR and Western blotting showed that transfection of TMEM88 siRNA downregulated the expression levels of PPAR-α and ACOX-1. Conversely, **(B)** pEGFP-C1-TMEM88 upregulated the expression levels of PPAR-α and ACOX-1. (The data are represented by at least three independent mean ± SD, *p < 0.05 vs normal group, ^#^ *p* < 0.05 vs control group).

To analyze the regulation mechanism of TMEM88 on lipid synthesis and metabolism cytokines, AML-12 cells were transfected with the TMEM88siRNA or pEGFP-C1-TMEM88. The results indicated that upregulation of TMEM88 inhibited the expression levels of SREBP-1c and FASN and upregulated PPAR-α and ACOX-1 in FFA-induced AML-12 cells (Figure 4A, Figure 5A). Meanwhile, the expression level of lipid synthesis and metabolism cytokines was opposite when TMEM88 was knocked out (Figure 4B, Figure 5B). All in all, these results indicated that the secretion of PPAR-α and ACOX-1 was positively regulated by TMEM88, while SREBP-1c and FASN were negatively regulated.

### TMEM88 Inhibited Cell Proliferation in Free Fat Acid–Induced AML-12 Cells

**FIGURE 6:**
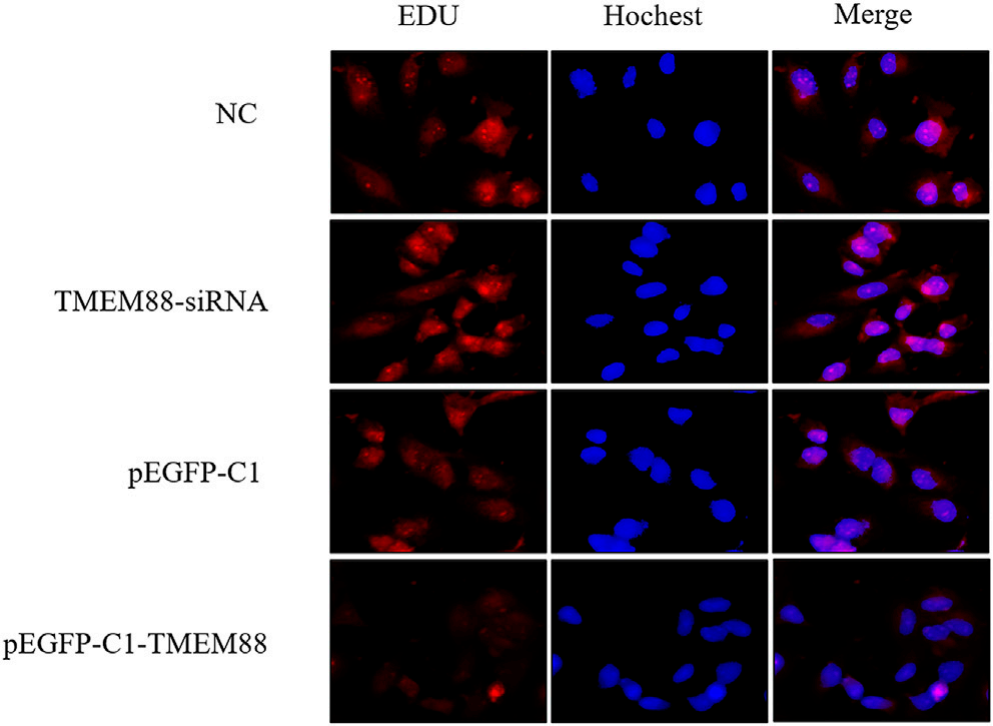
TMEM88 inhibited the proliferation of AML-12 cells stimulated by FFA. **(A)** Edu staining detected the effect of TMEM88 on the proliferation of AML-12 cells stimulated by FFA. The image of AML-12 cells stained by Edu (red) represents cell proliferation, and Hochest (blue) was used to mark the cell nucleus.

To explore the effect of TMEM88 on cell proliferation in FFA- induced AML-12 cells. Edu staining was used to detect proliferation. Cell reproduction was represented by the image of AML-12 cells stained with EDU (red), while the nucleus was marked with Hochest (blue). The results showed that TMEM88 siRNA can significantly promote the proliferation of activated AML-12 cells. On the contrary, the image indicated that the cell proliferation was inhibited after transfecting with pEGFP-C1-TMEM88 in FFA-induced AML-12 cells (Figure 6A).

### TMEM88 Promoted Cell Apoptosis in Free Fat Acid–Induced AML-12 Cells

**FIGURE 7:**
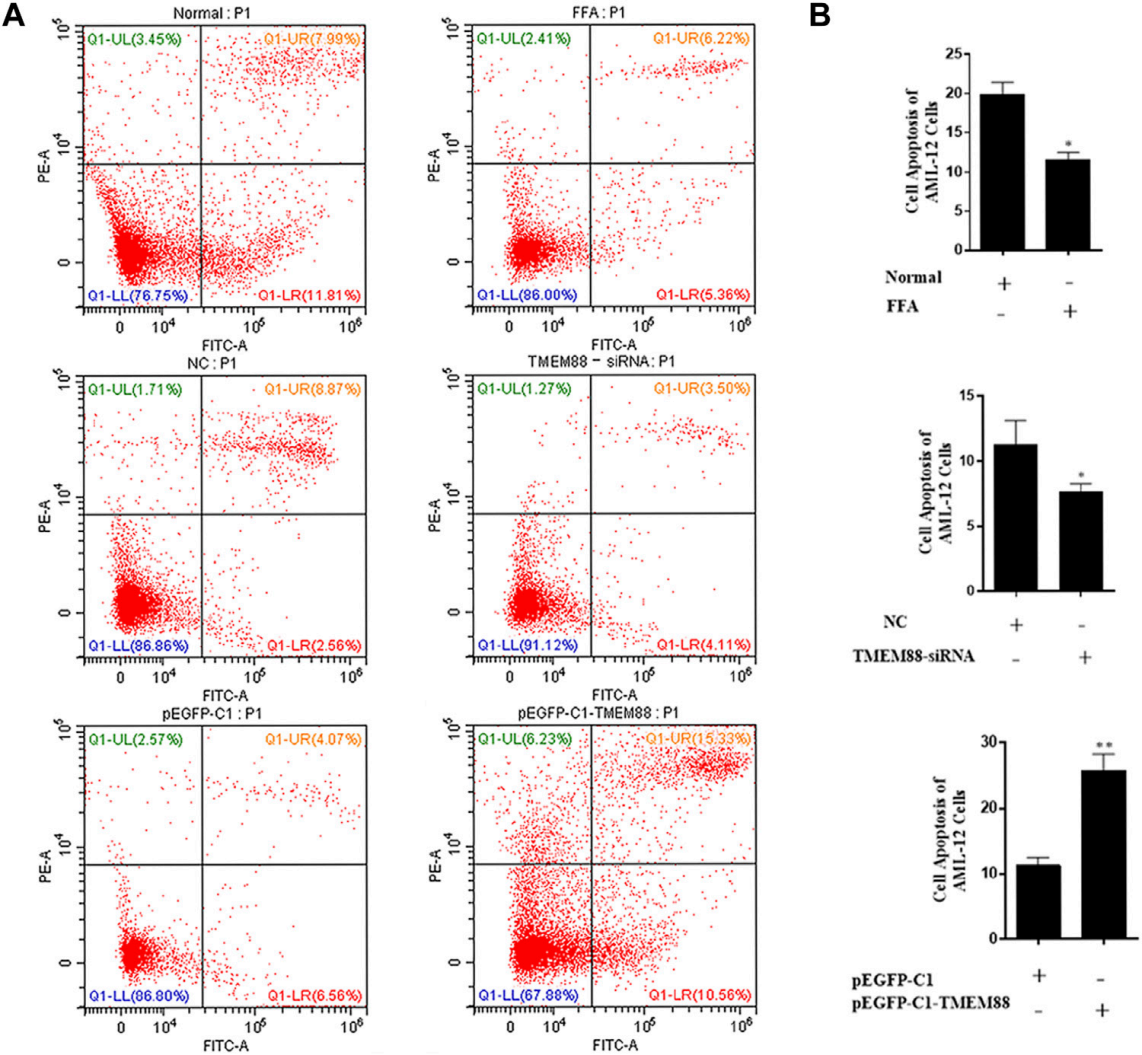
Apoptosis of AML-12 cells induced was inhibited by FFA. **(A,B)** Flow cytometer results indicated that the expression of TMEM88 was positively correlated with AML-12 cell apoptosis. (The data are represented by at least three independent mean ± SD, *p < 0.05, **p < 0.01 vs control group).

To research the function of TMEM88 on the cell apoptotic rate. Annexin V-FITC/PI double staining flow cytometry was used to process apoptosis. The result showed that the apoptosis of AML-12 cells was significantly inhibited when TMEM88 was knocked out. On the other hand, the overexpression of TMEM88 induced a higher apoptotic rate (Figures 7A,B). Above all, TMEM88 can regulate the apoptosis of AML-12 cells.

### TMEM88-Mediated Wnt/β-Catenin Signaling Pathway in Free Fat Acid–Induced AML-12 Cells

**FIGURE 8:**
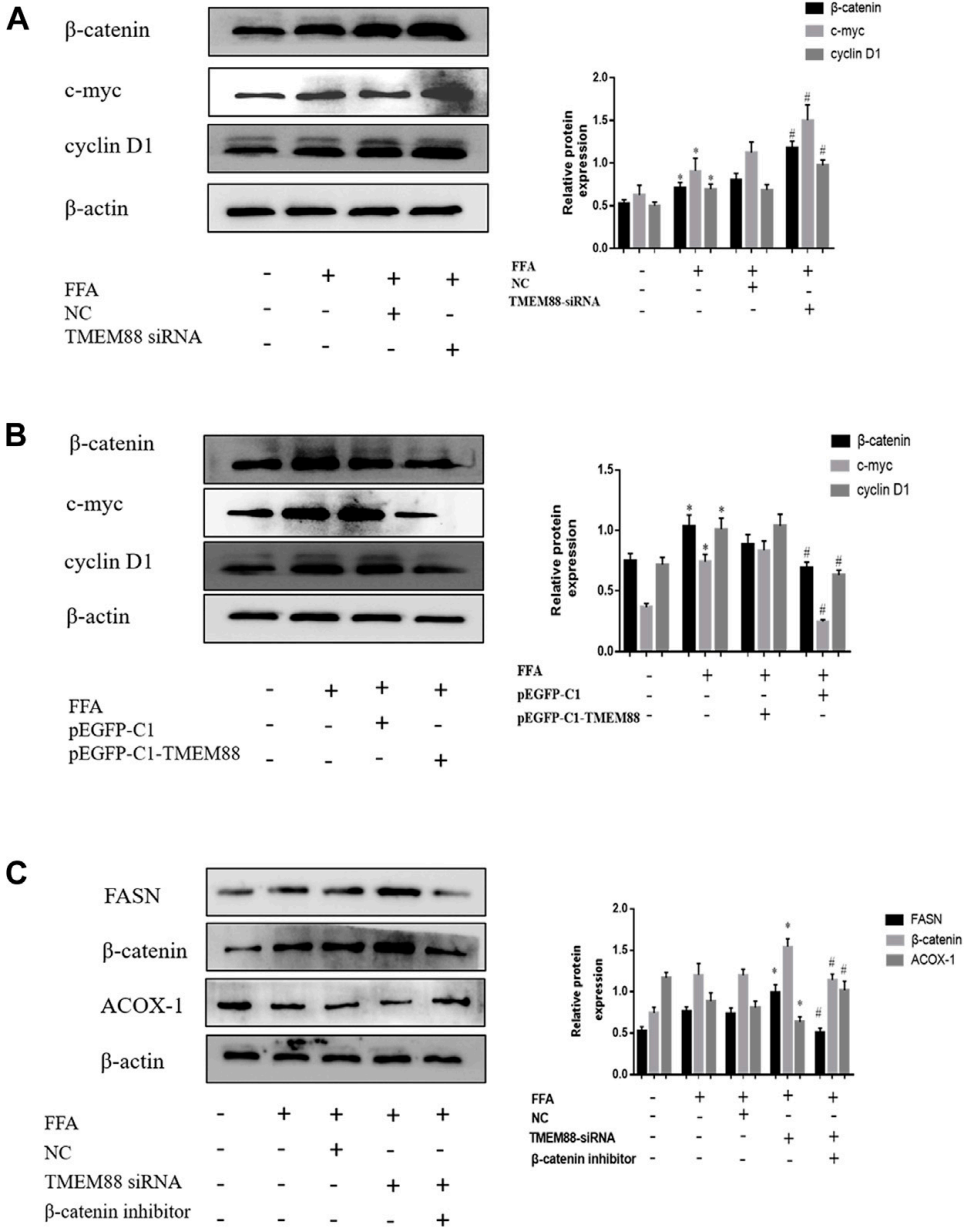
Expression of β-catenin and related signaling pathways in FFA-induced AML-12 cells. **(A,B)** The Wnt/β-catenin, c-myc, and cyclin D1 signaling pathways were elevated in FFA-induced AML-12 cells. **(C)** The downregulated of β-catenin can promote lipid metabolism. (The data are represented by at least three independent mean ± SD, *p < 0.05 vs control group, ^#^ *p* < 0.05 vs siRNA+β-catenin inhibitor group).

To detect the regulated mechanism of Wnt/β-catenin in NAFLD, this study found that the expression of the Wnt/β-catenin signaling pathway was increased in FFA-induced AML-12 cells. In addition, the Western blotting results suggested that the expression of β-catenin, c-myc, and cyclin D1 was greatly increased when knocking out TMEM88 (Figure 8A). On the other hand, β-catenin, c-myc, and cyclin D1 signaling pathways were significantly downregulated when TMEM88 was overexpressed in AML-12 cells (Figure 8B). Furthermore, the β-catenin inhibitor FH535 was used to inhibit the β-catenin signaling pathway. The result showed that the downregulation of β-catenin can promote lipid metabolism (Figure 8C). Briefly, these results showed that the Wnt/β-catenin signaling pathway may regulate the lipid metabolism of TMEM88 in AML-12 cells.

## Discussion

There is currently no effective treatment for NAFLD. Its pathogenesis is generally difficult to detect at the beginning. Its process includes fat accumulation, inflammatory infiltration, cell damage, fibrosis, oxidative stress, and cancerous, similar to alcoholic fatty liver (Cobbina and Akhlaghi 2017; Baselli and Valenti 2019; Hu et al., 2019). It is the main life-threatening reason for liver disease. The main feature is the accumulation of fat in the liver caused by lipid anabolism (Tian et al., 2016; Barbaro et al., 2018; Del Campo et al., 2018). Moreover, as the living environment changes, the incidence of non-alcoholic fatty liver disease (NAFLD) is increasing globally (Younossi et al., 2016; Zhou et al., 2019; Ray 2021). The prevalence of NAFLD worldwide is approximately 25% (Younossi et al., 2016), the lowest incidence in Africa is 13% (26707365 (Younossi et al., 2016), and the incidence in Southeast Asia is as high as 42% (Li et al., 2019). It is estimated that the prevalence of NASH in western Europe, the United States, Japan, and China will show an increase by 56% in the next 10 years (Huang et al., 2021). Furthermore, TMEM88 can inhibit the Wnt/β-catenin–mediated in inflammatory and cancer (Ma et al., 2017; Xu et al., 2018). This experiment explores whether the Wnt/β-catenin signaling pathway can be mediated by TMEM88 and then regulated lipid metabolism in NAFLD models.

Currently, the underlying molecular mechanism of lipid metabolism imbalance in liver cells is still not fully understood. In addition, the experiment indicated that TMEM88 also mediated the secretion of inflammatory cytokines, which showed that TMEM88 played a role in inflammatory diseases (Li et al., 2020). Moreover, the results of our past research showed that TMEM88 was reduced in HCC tissues and can be used to regulate HCC (Zhang et al., 2017). While TMEM88 in NAFLD has not been studied. The current research clarified the regulatory effect of TMEM88 in the fatty liver and its internal mechanism. First of all, protein and RNA results proved that the expression of TMEM88 was significantly reduced, compared with the normal group. The results demonstrated that TMEM88 may play an indispensable role in FFA-induced AML-12 cells. Furthermore, our research showed that in FFA-induced AML-12 cells and MCD-fed mice, the expression levels of SREBP-1c and FASN are higher in NAFLD, and ACOX-1 and PPAR-α expression levels were lower. Therefore, the experimental results demonstrated that the secretion of lipid metabolism cytokine SREBP-1c, PPAR-α, FASN, and ACOX-1 was regulated by TMEM88 *in vivo*. Moreover, this study also proved that the FASN and SREBP-1c were downregulated, while PPAR-α and ACOX-1 were increased by transfecting FFA-induced AML-12 cells with pEGFP-C1-TMEM88. Additionally, compared with the pEGFP-C1 transfection group, the FFA stimulation group and pEGFP-C1 transfection group were not statistically significant. In addition, the experiment proved that FASN and SREBP-1c were overexpressed, while the expression of PPAR-α and ACOX-1 was reduced by FFA-induced intracellular transfection with TMEM88 siRNA. Therefore, this experiment can infer that the baseline level of TMEM88 was essential for the expression of cell lipid metabolism cytokine genes. It can increase the level of lipid oxidation cytokines in AML-12 cells and negatively regulate lipid synthesis.

The classic Wnt/β-catenin signaling pathway is the best characterized Wnt pathway that participates in the development of animal embryos, and its dysfunction is closely related to a variety of other malignant tumors (Baselli and Valenti 2019). The β-catenin signaling pathway was phosphorylated and degraded by GSK3β when the Wnt signaling pathway was inhibited (Lee et al., 2021). In addition, the Wnt/β-catenin signaling pathway is the main target of the Hippo signaling pathway (Heallen et al., 2011). It also has the function of mediating liver fibrosis (Bhalla et al., 2012), liver regeneration (Cai et al., 2021), and HCC (Baselli and Valenti), and it can also influence the cell cycle (Barbaro, Romito, and Alisi). Studies showed that the Wnt/β-catenin signaling pathway can mediate tissue growth, development, homeostasis, and repair in a variety of ways (Majidinia et al., 2018; Fan et al., 2020). The Wnt/β-catenin signaling pathway was mediated by TMEM88 and then regulated the secretion of lipid metabolism cytokines. However, the molecular mechanism of the Wnt/β-catenin signaling pathway is still unclear in NAFLD HCC. There was no relevant research on the regulation effect of TMEM88 on the Wnt/β-catenin signaling pathway. Furthermore, hepatic steatosis needed further research. The previous research reports showed that liver fibrosis can be reduced by reversing the activation of HSCs and accelerating hepatocyte apoptosis by blocking the Wnt/β-catenin signaling pathway (Chen et al., 2020; Huang et al., 2021; Jing et al., 2021). However, the relationship between the Wnt/β-catenin signaling pathway and NAFLD has not been reported yet. Therefore, this study deduced the key pathway of TMEM88 regulation mechanism *in vivo*. Moreover, the study result proved that the expression of the Wnt/β-catenin signaling pathway was decreased after FFA-induced AML-12 cells transfected with the TMEM88 overexpression plasmid. In conclusion, research results showed that the influence of intracellular lipid metabolism cytokines by Wnt/β-catenin, cyclin D1, and c-myc were inseparable from the mediation of TMEM88 in FFA-induced AML-12 cells.

In summary, this study showed that in FFA-induced AML-12 cells, the TMEM88 transmembrane protein and the Wnt/β-catenin signaling pathway can mutually regulate the secretion of cell lipid metabolism cytokines. In the next experiment, transcriptome sequencing will be performed on FFA-stimulated AML cells to find upstream regulatory cytokines of TMEM88, such as RNA or DNA. It is a great task to master the principle of these lipid metabolism regulators, but we must explore these regulators as a breakthrough point in the treatment of NAFLD-related diseases. Furthermore, with the continuous in-depth research on NAFLD and the development of science and technology, it is believed that the pathogenesis of NAFLD can be better clarified in the near future, a target for the treatment of NAFLD can be explored, and more patients can be cured.

## Data Availability

The original contributions presented in the study are included in the article/supplementary material; further inquiries can be directed to the corresponding authors.
